# The Early Recognition of Mental Diseases in Private Practice

**Published:** 1905-09-02

**Authors:** 


### THE EARLY RECOGNITION OF MENTAL DISEASES IN PRIVATE PRACTICE.

The July issue of the *West London Medical Journal* contains a paper on this subject by Dr. W. H. B. Stoddart. The author is of opinion that the generality of practitioners are not sufficiently on the look-out for early evidences of mental instability, and that this oversight not infrequently accounts for loss of invaluable time, if not for many of the tragedies recounted in the daily Press. He thinks that the neglect above mentioned depends in part upon the customary tendency to fix the professional eye upon lapses from physical health, in part upon the wealth of euphemisms with which conditions bordering upon, if not actually transgressing the boundaries of, insanity are habitually minimised by the profession. Thus "hysteria," "anorexia nervosa," "nerves," "neurasthenia," and even "liver" may occasionally be made the stumbling-block over which treatment may loiter until the early stages of the disorder, correct treatment of which is vital to success, have been idly squandered. When a young girl takes to drinking her urine and screaming the house down several times a day, she is not hysterical, she is insane; so, too, a patient who feels tired of life and wishes to be always alone and refuses food is not neurasthenic, but insane to a mild extent, and probably melancholic. A good instance of this misinterpretation of early warnings of disordered mental equilibrium is provided by the following case, related by the author. A man of 35 observed that he felt ill and depressed on retiring to his bedroom of an evening, and reached the conclusion that poisonous vapours were being instilled into his room. He consulted his doctor on the subject, and was advised to change his quarters. The patient next laid an information with the police, who examined the room, and subsequently removed the patient to an infirmary, for the treatment of his delusion. Dr. Stoddart observes that the police have more knowledge of mental disorders than have many gentlemen of the medical profession. He thinks that the standard of physical health set up as normal by medical men generally is a far higher one than the standard of mental health which they are prepared to accept as being within the limit of variation of sanity. Thus the patient who has indigestion, or a relaxed throat, or an acne pustule, is likely to meet with a careful examination and active treatment, while the man who complains that he is suffering from depression of spirits is lightly dismissed, often without examination, and not seldom forgotten until an attempt at suicide affords a vivid explanation of his symptoms. So again with loss of memory, which may easily be dismissed with a few cheerful remarks, and may yet be the premonitory symptom of a fatal general paralysis.

Dr. Stoddart gives several practical hints of importance upon the best course of treatment in private practice. He insists on the unwisdom of using such ill-omened words as "mad," "lunatic," or "asylum," which throw difficulties in the way of treatment on sentimental grounds. Relatives of patients who would have no objection to their treatment in a private home, evince a marked and natural antipathy to the idea of their being "put away in a mad-house." This becomes of importance because, in the author's opinion, the majority of cases are far better treated in institutions than at home. As regards travel, he observes that in such incurable insanities as dementia, travel is a mere waste of money, while in the acute and curable insanities travel cannot be too strongly condemned. The same applies to manual labour which is often recommended. Both these means are of value after recovery, as an aid to the completion of the cure, but quite inadmissible during the acute stages of insanity. The best cases for private treatment at home are patients suffering from dementia, chronic quiet delusional cases, and some forms of obsession. As an instance of the latter class the author quotes a curate of his acquaintance whose obsession is that he has pushed some one into the ink-pot. He knows the absurdity of the idea, and by a mental effort can refrain from looking into the ink-pot to make certain, but presently the obsession returns with added force, he gratifies it by a glance, and is then able to progress peacefully with whatever work he has in hand. For cases such as these the greater variation of home life, as opposed to that in institutions, makes the former preferable.

But supposing for any reason a fairly severe case has to be treated at home, asylum nurses, male or female according to the sex of the patient, are absolutely necessary, preferably those holding the certificate of the Medico-psychological Association. If force has to be occasionally employed, a sufficiency of attendants is of great importance, in order that bodily injuries to the patient may be avoided. The room chosen should be on the ground floor; the bedstead should be removed and the bed laid upon the floor; the window should be boarded up below and not allowed to open more than 9 inches above; anything that can be converted to a dangerous use must be taken away, and the room as far as possible padded with mattresses if the patient be very violent. In the matter of medicinal treatment, all symptoms such as anæmia should be dealt with by appropriate drugs; purgation is an important help, for such patients are often constipated, although they may have a symptomatic diarrhœa. Dr. Stoddart holds that the best hypnotics for the sane are bromides and hyoscyamus, chloralamide, trional and veronal, but that they are of little value in insane states. For the latter he advises sulphonal, paraldehyde, and amylene hydrate, paraldehyde being particularly indicated by a difficulty in falling to sleep, sulphonal by a tendency to awake too early. A general anæsthetic may be necessary for the induction of sleep in acute delirious mania. The case is instanced of a patient who had not slept for five days and nights, and had taken no food for 48 hours. She was so violent that nothing could be done for her, and was naturally becoming exhausted. She was anæsthetised with chloroform, the stomach washed out and some food given by the stomach tube, and the lower bowel cleared by an enema. The anæsthetic was then continued for an hour, after which the patient slept for 16 hours. She took food on waking, and was convalescent in a fortnight.

